# Identification of aging‐associated immunotypes and immune stability as indicators of post‐vaccination immune activation

**DOI:** 10.1111/acel.13703

**Published:** 2022-09-08

**Authors:** Alper Cevirgel, Sudarshan A. Shetty, Martijn Vos, Nening M. Nanlohy, Lisa Beckers, Elske Bijvank, Nynke Rots, Josine van Beek, Anne‐Marie Buisman, Debbie van Baarle

**Affiliations:** ^1^ Center for Infectious Disease Control National Institute for Public Health and the Environment Bilthoven The Netherlands; ^2^ Department of Medical Microbiology and Infection prevention Virology and Immunology Research Group University Medical Center Groningen Groningen The Netherlands

## Abstract

Immunosenescence describes immune dysfunction observed in older individuals. To identify individuals at‐risk for immune dysfunction, it is crucial to understand the diverse immune phenotypes and their intrinsic functional capabilities. We investigated immune cell subsets and variation in the aging population. We observed that inter‐individual immune variation was associated with age and cytomegalovirus seropositivity. Based on the similarities of immune subset composition among individuals, we identified nine immunotypes that displayed different aging‐associated immune signatures, which explained inter‐individual variation better than age. Additionally, we correlated the immune subset composition of individuals over approximately a year as a measure of stability of immune parameters. Immune stability was significantly lower in immunotypes that contained aging‐associated immune subsets and correlated with a circulating CD38 + CD4+ T follicular helper cell increase 7 days after influenza vaccination. In conclusion, immune stability is a feature of immunotypes and could be a potential indicator of post‐vaccination cellular kinetics.

## Graphical abstract

We identified nine phenotypically distinct immunotypes that are allowed improved description of inter‐individual variation compared to age, despite retaining age‐associated signatures. We observed that CMV+ and older individuals showed greater immune variation. Moreover, immune stability positively correlated to an increase in CXCR5+ CD38+ CD4+ T cells 7 days after influenza vaccination, negatively correlated to age, and was immunotype‐dependent.

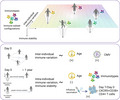

## 1 INTRODUCTION

The immune system is constantly remodeled throughout life by internal and external factors (Simon et al., 2015). Alterations in the immune system caused by these factors accumulate over time and contribute to highly heterogeneous immunological states observed in the aging population (Liston et al., 2021). These alterations could lead to a decline in immune system functionality at older age, known as immunosenescence, which is associated with increased susceptibility to infections and reduced vaccination responses (Fulop et al., 2020). Vaccination is an effective measure to protect the aging population from infections (Andre et al., 2008). However, as vaccination responsiveness varies between individuals, particularly in older adults, identifying those who do not respond to vaccines and why is essential to design alternative vaccination strategies (Lang et al., 2012) or immunomodulatory interventions (Aiello et al., 2019) to improve protection of these individuals. We postulate that immunological characteristics, amongst which immune cell subset composition, play an essential role in immune functionality. To this end, we need to better understand immune variation in the aging population, as well as the key phenotypical features and functional capacities of these immune subset compositions.

Several studies have investigated age‐related changes in the immune system and immune variation based primarily on the percentages of immune cell subsets and serum proteins (Brodin et al., 2015; Carr et al., 2016). These studies showed that immune variation was primarily driven by non‐heritable influences (Brodin et al., 2015) and the immune cell subset compositions were shaped by age and cohabitation (Carr et al., 2016). In addition, a number of studies sought to define immunotypes based on combinations of specific immune cell subsets to explain the immune variation and describe the immunological characteristics of immune subset compositions as immunotypes (Kaczorowski et al., 2017; Kanodia et al., 2019; Liefferinckx et al., 2021). However, these studies did not reveal discrete immunotypes, which could be due to immune parameters investigated, clustering methods utilized, and the characteristics of the study population. Identification of immunotypes in the population is essential to understand immune variation and to pinpoint individuals at risk of mounting lower/no response to vaccines.

Here, we analyzed both percentages and absolute numbers of immune cell subsets in healthy individuals to describe age‐related changes in the immune system and factors associated with immune variation. We also measured the seropositivity for chronic herpes virus infections: Cytomegalovirus (CMV) and Epstein–Barr virus (EBV) since they are responsible for a broad modulation in immune cell subset signatures (Albanese et al., 2017; Klein & Flanagan, 2016; Loewendorf & Benedict, 2010; Wang et al., 2014). Based on the immune cell subset composition of young (25–49 years), middle‐aged (50–64 years), and older adults (65–92 years), we characterized discrete immunotypes that could explain immune variance superior to chronological age. Additionally, we defined immune parameters that are associated with these immunotypes. Furthermore, we compared immune cell subset (percentage and absolute number) compositions across approximately 1 year to quantify the intra‐individual immune variation. We observed that immunotypes that exhibited signatures of aging‐related immune subsets had significantly higher intra‐individual variation. Furthermore, we show that longitudinal intra‐individual variation, which reflects immune stability, was associated with circulating CD38+ CD4+ T follicular cell kinetics 7 days after influenza vaccination. Overall, our results suggest that the immune parameters identified in immunotypes and stability of immune parameters could help us toward improved characterization of individuals at risk of mounting low vaccination responses.

## 2 RESULTS

### 2.1 Study population characteristics

**FIGURE 1:**
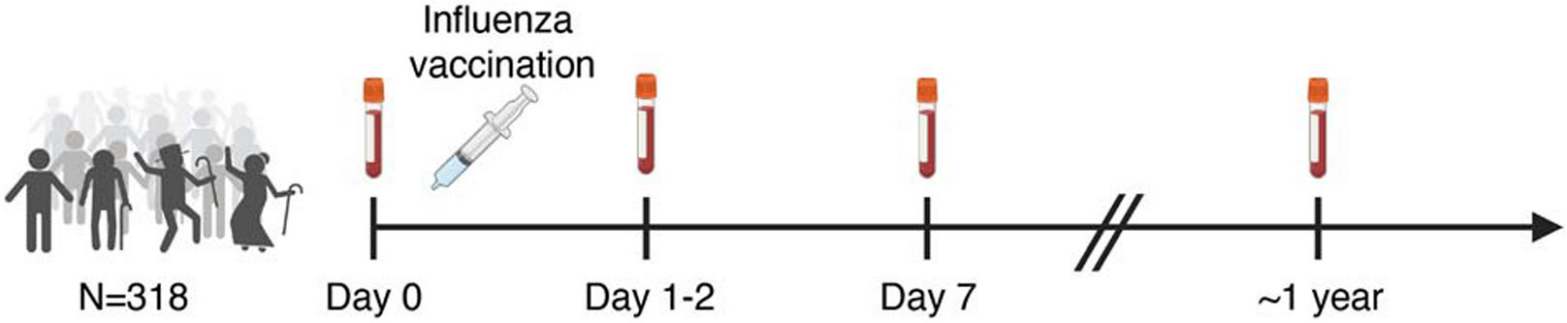
VITAL vaccination study design

To better understand immune decline with age and changes in immune subsets after influenza vaccination, we analyzed immune cell subsets in blood samples of 318 individuals (for whom EDTA blood was available), out of 326 individuals participating in the vaccines and infectious diseases in the aging population (VITAL) cohort (van Baarle et al., 2020) (Figure S1). Whole blood samples from these individuals were analyzed for a total of 103 (Table S1) innate and adaptive immune cell subsets (percentage and a absolute number) by flow cytometry before (Day 0) and 1–2 days, 7 days, and approximately 1 year after influenza vaccination (Figure 1). Individuals excluded from the analysis are described in the flow chart (*N* = 15, Figure S1). The characteristics of the 303 remaining cohort participants are summarized in Table 1. Young adults comprised a higher percentage of females than older adults. No difference in seropositivity for the chronic herpesvirus CMV and EBV was identified between age groups (chi‐squared test, *p*‐value>0.05). Based on the leukocyte data obtained from DxH 500 hematology analyzer, the older adults had significantly higher absolute numbers of white blood cells (WBC), monocytes (MO), neutrophils (NE), and they had lower percentages of lymphocytes (LY) than middle‐aged and young adults. MO and NE percentages were significantly higher in older adults than younger adults (Table 1). There were no significant immune subset differences observed between young and middle‐aged adults.

**TABLE 1:** Characteristics of VITAL cohort participants

	Young adults (YA) *N* = 60	Middle‐aged adults (MA) *N* = 89	Older adults (OA) *N* = 154	*N* = total	Comparison age group	*p*‐Value
Age in years	35 (25, 49)	58 (50, 64)	75 (65, 92)	303		NT
Female % (*N*)	67% (40)	58% (52)	47% (73)	303	YA vs OA	0.033
CMV+ % (*N*)	38% (23)	49% (44)	51% (78)	303		ns
EBV+ % (*N*)	77% (46)	89% (79)	84% (130)	303		ns
WBC counts 10^9^/L	5.59 (2.87, 10.99)	5.62 (2.77, 16.67)	6.32 (3.41, 11.65)	301	YA vs OA MA vs OA	0.007 0.002^*^
LY counts 10^9^/L	1.68 (1.11, 3.87)	1.65 (0.74, 3.94)	1.66 (0.73, 4.21)	301		ns
MO counts 10^9^/L	0.39 (0.18, 0.80)	0.40 (0.15, 1.16)	0.49 (0.23, 0.95)	301	YA vs OA MA vs OA	<0.001 <0.001^*^
NE counts 10^9^/L	3.26 (1.41, 7.10)	3.22 (1.66, 12.71)	3.74 (0.75, 8.38)	301	YA vs OA MA vs OA	0.004 0.004
LY %	30 (20, 53)	30 (16, 49)	27 (10, 58)	301	YA vs OA MA vs OA	<0.001 0.019
MO %	7.13 (4.22, 10.77)	7.33 (3.68, 12.38)	7.86 (3.59, 20.90)	301	YA vs OA	0.004
NE %	59 (33, 72)	60 (42, 79)	63 (19, 80)	301	YA vs OA	0.018

*Note*: Age and leukocyte numbers are medians per group and ranges in parantheses. For categorical variables, *p*‐values obtained from χ^2^ test, for continuous variables *p*‐values obtained from Kruskal–Wallis test (both followed by Dunn's test when *p*‐values are significant, Benjamini–Hochberg corrected) are reported. Significant comparisons between age groups are stated in comparison age group column.

Abbreviations: NT = not tested, ns = *p* > 0.05 not significant.

### 2.2 Relationship between age and immune cell subset percentages and absolute numbers

**FIGURE 2:**
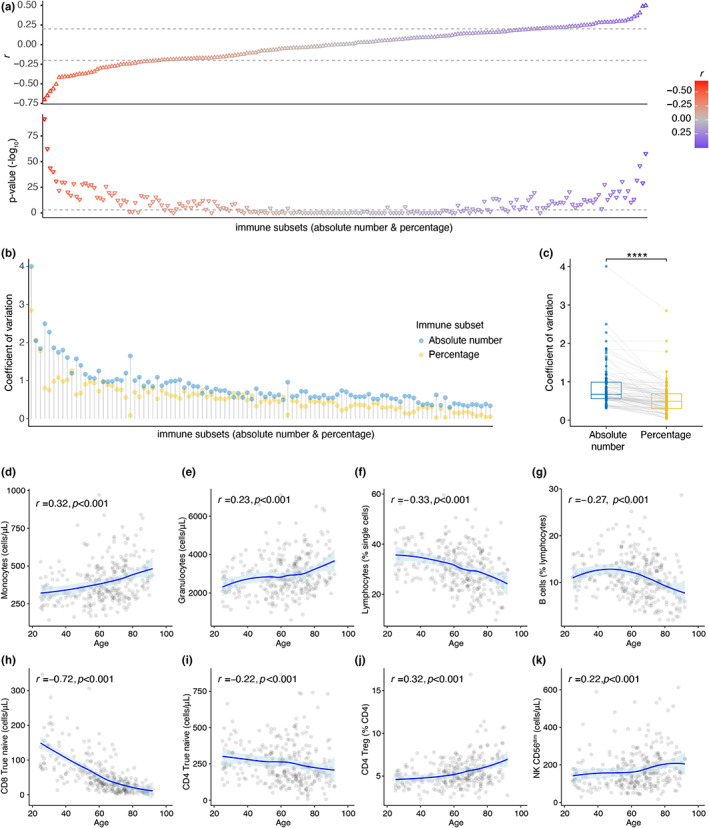
Age strongly correlates to immune cell subset percentages and absolute numbers. (a) Spearman correlations between age and immune cell subsets (flow cytometry immune phenotyping, percentage and absolute number). Spearman correlation coefficients (*r*) and *p*‐values (Bonferroni correction) are reported. Color gradient represents *r*. Dashed lines present the cut offs (−0.2 and 0.2 for *r*, 0.001 for *p*‐value). (b) Coefficient of variation for immune cell subset percentages and absolute numbers. The coefficient of variation is calculated for each immune subset across the cohort as a ratio of standard deviation to mean. (c) Comparison of coefficient of variances between percentage and absolute number of each immune cell subset (Wilcoxon signed‐rank test). (d–k) Spearman correlations between selected immune cell subsets and age are plotted. The area around the loess fitted regression line describes 95% confidence interval (*p*‐values are not false discovery rate corrected)

We investigated the relationship between age and both pre‐vaccination percentages and absolute numbers of 103 immune cell subsets obtained by flow cytometry immunophenotyping. After we applied a threshold of *p*‐value ≤0.001 (Bonferroni corrected) and Spearman correlation coefficient (*r*) of *r* ≤ −0.2 or *r* ≥ 0.2, we observed that the number of significant correlations with age was lower for immune cell subset percentages (*n* = 32) than absolute numbers (*n* = 42) (Figure 2a, Table S1). In addition, absolute numbers of immune cell subsets were significantly more heterogeneous between individuals than percentages of immune cell subsets (Figure 2b,c). To investigate which subsets were more heterogeneous at younger and older age, we built linear regression models for each immune subsets using age, sex, and CMV‐seropositivity and correlated the residuals of these linear regression models with age. We observed that CD8 + CD38 + HLA‐DR+ (absolute numbers) and CD8 + CD38+ T cells (absolute numbers) were more heterogeneous at younger age, whereas CXCR5 + CD4+ Teff cells (absolute numbers) and CXCR5 + CD8+ Teff cells (absolute numbers) were most heterogeneous at older age (Table S2).

Age‐related changes in the immune system affect both innate and adaptive immune compartments. In the innate myeloid cell compartment, consistent with previous reports (Pang et al., 2011), we observed increased numbers of monocyte and granulocyte with higher age (Figure 2d,e). In the adaptive lymphoid cell compartment, in line with the age‐dependent reduction in thymic output and bone marrow activity (Palmer, 2013), total lymphocytes, B cells, true naive (Gattinoni et al., 2011, 2017) (CD45RO‐CD27 + CCR7 + CD127 + CD95‐) CD8+ T cells, and true naive CD4+ T cells exhibited an inverse relationship with age (Figure 2f–i). In contrast to true naive CD8+ T cell numbers (*r* = 0.72, *p* < 0.001), the age association in true naive CD4+ T cell numbers (*r* = 0.22, *p* < 0.001) was less pronounced. Another critical cell type known to be altered with age is regulatory T cells (Treg) (Johannes Fessler et al., 2013). Indeed, CD4+ Treg percentages correlated positively (*r* = 0.32, *p* < 0.001) with higher age (Figure 2j). In the Natural Killer (NK) cell compartment, age positively correlated with absolute numbers of CD56^dim^ NK cells (*r* = 0.22, *p* < 0.001) (Figure 2k), which are the highly cytotoxic NK cells, and negatively correlated with percentages of CD56^bright^ NK cells (*r* = −0.2, *p* < 0.001, Table S1) which are a major cytokine‐producing (Jacobs et al., 2001, p.56) subset. However, absolute numbers of CD56^bright^ NK cells did not correlate with age (*r* = −0.04, *p* = 0.10, Table S1). Additionally, we investigated HLA‐DR expression on T & NK cells. HLA‐DR+ CD8+ Tregs were described to have a regulatory function similar to classical CD4+ Foxp3+ Tregs (Machicote et al., 2018, p.8). HLA‐DR+ CD4+ Tregs were shown to have increased regulatory function than HLA‐DR‐ CD4+ Tregs (Schaier et al., 2013). We observed that the HLA‐DR+ percentage of CD4+, CD8+, CD4+ Treg, and CD56^dim^ NK cells correlated with age (Figure S2B).

Interestingly, we noticed that some immune subsets we reported above demonstrated non‐linear age‐related patterns, which means stronger negative or positive associations in different age groups (Figure S2A,B). Furthermore, when we ordered individuals by increasing age on a heatmap, we observed that immune cell subsets percentages were highly heterogeneous between individuals with similar ages (Figure S2C). Therefore, investigation of age‐related changes in immune cell subsets one at a time imposes a major limitation to our understanding of the aging of the immune system.

### 2.3 Substantial inter‐individual immune variation

**FIGURE 3:**
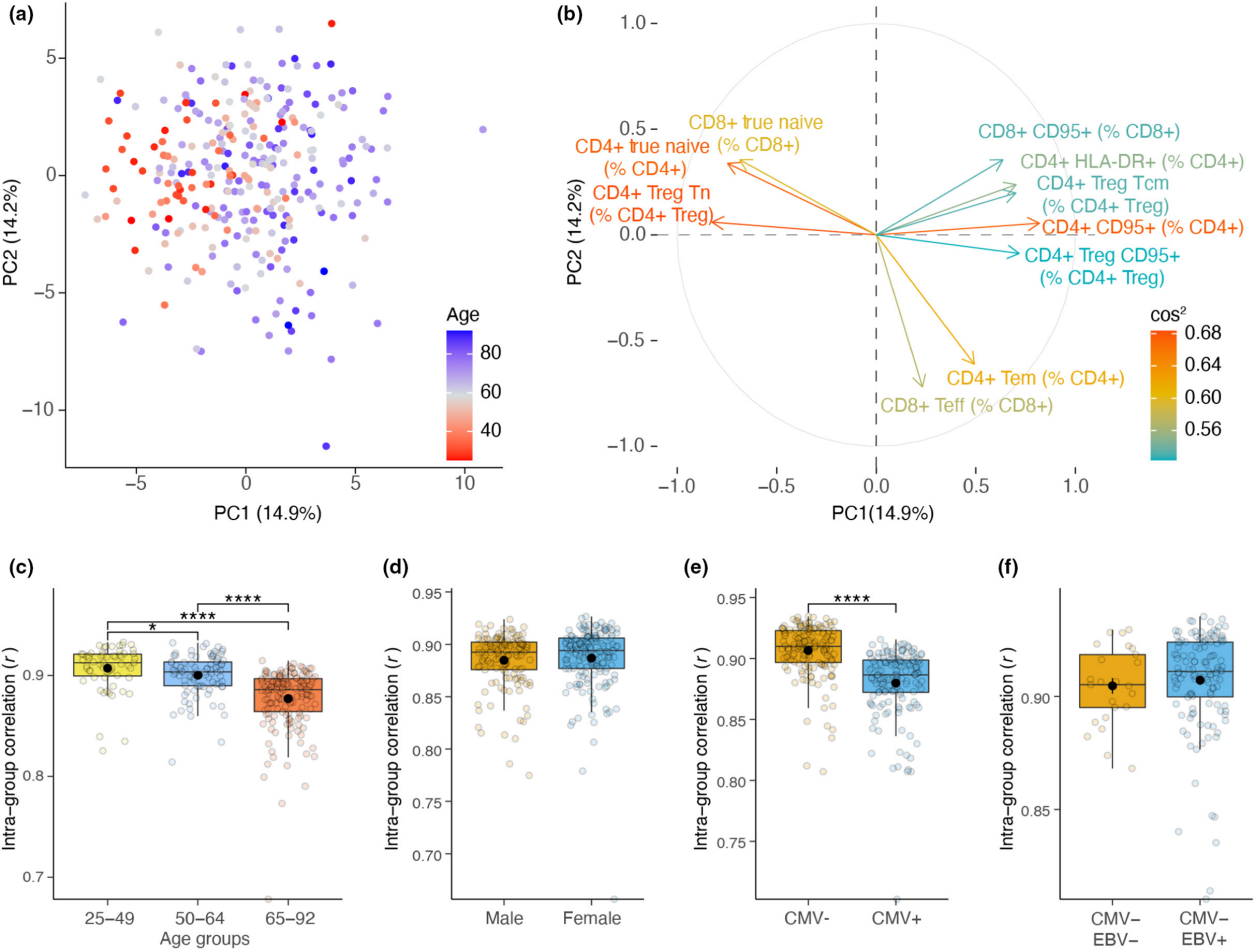
Age and CMV‐seropositivity is associated with increased inter‐individual immune variation. (a) Projection of 59 immune cell subsets (percentage) with minimal overlap onto the two Principal Components (PC). (b) Contribution of immune cell subsets on PCs. Top 10 contributing immune cell subsets are colored by their square of cosine (cos^2^). The higher cos^2^ is, the higher contribution of variable on PCs. (c) Comparison of intra‐group similarity (spearman correlation) in age groups, (d) sex differences, (e) CMV‐seropositivity, and (f) EBV‐seropositivity based on percentage and absolute number of immune cell subsets (Dunn's test, Benjamini–Hochberg corrected *p*‐values)

In order to identify the immune cell subsets that may explain immune variation between individuals in our study cohort, we used a dimensionality reduction method: Principal Component (PC) analysis. We selected 59 immune cell subsets with minimal overlap that represent the diverse features of the innate and adaptive immune network (Materials and Methods, Table S1) and projected these immune cell subsets onto PCs. PC1 and PC2 explained 14.9% and 14.2% of the total variance, respectively (Figure 3A). We observed an underlying age gradient on PC1, which suggests that immune subsets represented on PC1 were influenced by age (Figure 3A). Nevertheless, this age gradient was not continuous, and individuals from different age groups were observed at a close distance. To further investigate the distribution of individuals across PCs, we plotted the top 10 immune subsets that contributed to PC1‐2 variation most. We observed that mostly younger individuals resided on the left‐hand side of PC1 owing to their high naive T‐cell subset proportions. In contrast, a higher proportion of older individuals who shared signatures associated with HLA‐DR+ and CD95+ expressing T cells were detected on the right‐hand side of PC1 (Figure 3B). In addition, CD8+ T effector (Teff) cells showed the highest contribution to PC2, which suggests that CMV‐seropositivity, which highly contributes to this subset (Derhovanessian et al., 2011; Karrer et al., 2003), could be more associated with the immune variation on PC2 (Figure 3b).

In addition, we observed that older adults were more spread across PCs than young adults, which suggests a higher inter‐individual variation in immune cell subset composition (Figure 3a). Therefore, we investigated the relationship between age and inter‐individual immune variation using intra‐group Spearman correlations in age groups. In older age groups, intra‐group correlations were significantly lower, which confirmed that heterogeneity in immune cell subset compositions increases with chronological age (Figure 3c). Furthermore, CMV‐seropositivity significantly contributed to the observed immune variation (Figure 3e); in contrast, sex or EBV‐seropositivity did not (Figure 3d,f). Lastly, we used permutation analysis of variance (PERMANOVA) to calculate the overall inter‐individual variance explained (R^2^). Sex, CMV‐seropositivity, and EBV‐seropositivity accounted for limited degrees of total variance; 2%, 5%, and 1%, respectively (Figure S3A–C). Similarly, only 5% of the total variance (PERMANOVA, *p* < 0.001) was explained by age. In addition, we observed specific cell subsets to be related to sex, CMV‐seropositivity, and EBV‐seropositivity. Males displayed lower naive T cells than females (Figure S3A). CD4+ Teff, CD8+ Teff, and CXCR5+ CD8+ Teff cells were higher in CMV+ individuals (Figure S3B). Lastly, we observed lower CXCR5+ CD8+ T naive cells in EBV + CMV‐individuals than EBV‐CMV‐ (Figure S3C). Thus, these results highlight that age could inadequately explain immune variation based on the immune parameters investigated. In addition to age, factors including sex, CMV‐seropositivity, and EBV‐seropositivity contributed to explaining an additional degree of immune variation.

### 2.4 Unique immune cell subset compositions displayed by immunotypes

**FIGURE 4:**
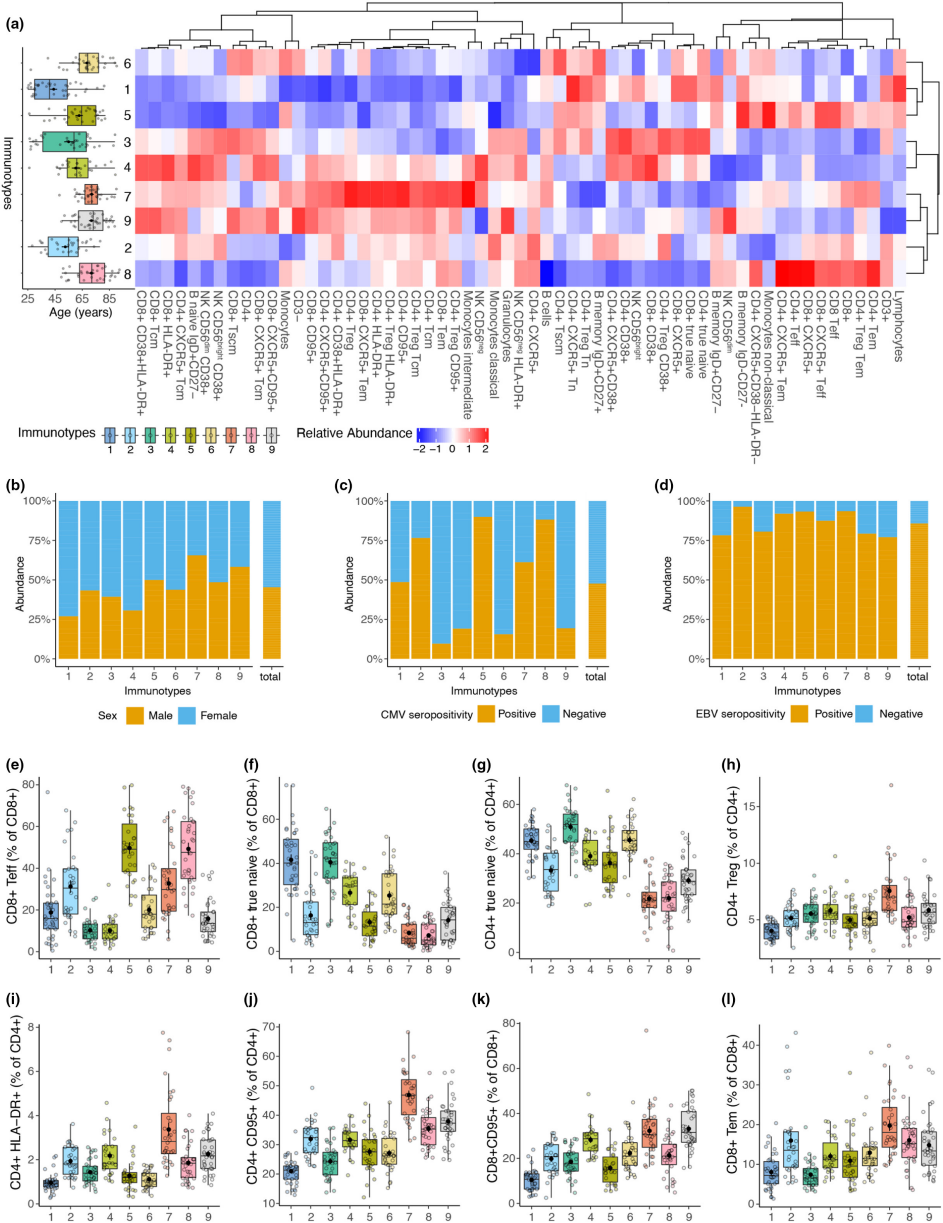
Immunotypes display immune cell subset compositions associated with CMV‐seropositivity and signatures of aging‐associated immune subsets. (a) Boxplot displays age distribution in immunotypes. Immunotypes are ordered by increasing median age. Heatmap shows percentages of 59 immune cell subsets in immunotypes. (b–d) sex, CMV‐seropositivity, and EBV‐seropositivity in immunotypes. Borderline CMV and EBV cases were removed from the plots. (e–i) Boxplots depicting selected immune cell subsets in immunotypes. Significant differences of immune cell subsets between immunotypes are reported in Table S2 (Dunn's test, Benjamini–Hochberg corrected *p*‐values)

To better explain the inter‐individual immune variation, we aimed to classify individuals based on their similarities of immune subset compositions. To this end, we applied gap statistics and k‐means clustering, which suggested that individuals could be grouped into nine immunotypes displaying unique immune subset compositions (Figure S4A–C, Figure 4a). These immunotypes explained 19% of the total immune variance (PERMANOVA, *p* < 0.001) compared with 5% variance explained by age (PERMANOVA, *p* < 0.001).

To describe these immunotypes, we investigated factors that may influence them and their specific composition. Key immune cell subset signatures of the identified immunotypes are described in Table 2. No effect of sex and EBV‐seropositivity was found; however, CMV‐seropositivity greatly attributed to a distinction between immunotypes and was 90% and 86% in immunotypes 5 and 8, respectively (Figure 4b–d). As expected, immunotypes with CMV+ individuals contained the highest proportions of CD4+ and CD8+ Teff cells (Figure 4a,e). Furthermore, CMV‐seropositivity was inversely associated with CD4+ and CD8+ true naive T‐cell percentages (linear regression, corrected for age and sex, *p* < 0.001), resulting in low CD4+ and CD8+ true naive T cell abundances in immunotypes 2 and 5 (Figure 4f–g).

**TABLE 2:** Key immune cell subset signatures in immunotypes

Immunotype	CMV+ (%)	Immune subsets (high)	Immune subsets (low)
1	49%	CD8+ True naive	CD4+ Treg, CD4+ CD95+, CD8+ CD95+, CD4+ Tcm, CD8+ Tcm
2	77%	Classical monocytes, CD4+ HLA‐DR+, CD4+ Tcm, CD8+ Tem	CD8+ True naive
3	10%	CD8+ True naive, classical monocytes, CD4+ Tscm	CD4+ CD95+
4	19%	CD8+ True naive, CD4+ HLA‐DR+, CD4+ Tcm	CD4+ Treg
5	90%	CD4+ Tscm	CD8+ True naive, classical monocytes, CD4+ HLA‐DR+, CD4+ Tcm, CD8+ Tcm
6	16%	CD8+ True naive, CD4+ Tscm, CD8+ Tscm	CD4+ HLA‐DR+, CD4+ Tcm
7	61%	CD4+ Treg, CD4+ CD95+, CD8+ CD95+, CD4+ HLA‐DR+, CD4+ Tcm, CD8+ Tem	CD8+ True naive
8	88%	CD19+ CD95+	CD8+ True naive, CD4+ Tscm, CD8+ Tscm
9	19%	CD8+ CD95+, CD8+ Tscm, CD4+ Tcm, CD19+ CD95+	CD8+ True naive

Immunotypes 1 and 3, which represent younger adults, showed the highest CD4+ true naive and CD8+ true naive T‐cell percentage (Figure 4f–g). In addition, despite containing older individuals, immunotype 6 showed similar CD4+ true naive T‐cell percentages to immunotypes 1 and 3 (Figure 4f–g). We observed that the participants in the interquartile range for immunotypes 7, 8, and 9 (median age > 70) contained a more narrow age range than those in immunotypes 1–2 (median age ≤ 55) (Figure 4a). Thus, the absence of younger adults in these immunotypes could indicate that these immune phenotypes could result from a highly remodeled immune system. Low percentages of true naive CD4+ T cell and naive CD4+ Treg considered as signs of a highly remodeled and older immune system (Churov et al., 2020) were the lowest in immunotypes 7, 8, and 9 (Figure 4a,g). Although immunotype 2 contained much younger individuals than immunotypes 7–9, immunotypes 2, 7, 8, and 9 were closely related based on correlation distances on the heatmap (Figure 4a). In addition, we investigated immune subsets that express CD95 which was recently shown to elicit non‐apoptotic signals and promote inflammation (Gallo et al., 2017). Additionally, CD95 expression is observed in memory T cells (Gattinoni et al., 2017). We observed that immunotypes 7, 8, and 9 showed significantly higher CD95+ CD4+, CD95+ CD8+, HLA‐DR+ CD4+, and CD8 Tem cell percentage than immunotypes 1 and 5 (Figure 4a, i–l). Interestingly, percentages of HLA‐DR+ CD4+, CD4+ Treg, HLA‐DR+ CD4+ Treg, CD95+ CD4+, and CD8 Tem cells were most abundant in immunotype 7 (Figure 4a,h–j,l). Lastly, the CD8+ T stem cell‐like memory (Tscm) to true naive CD8+ T cell ratio was significantly higher in immunotype 7 than immunotypes 1–6 (Figure S4D, Table S3).

In summary, we hypothesize that immunotype 6 could be a candidate immunotype that includes older but immunologically fit individuals. In contrast, immunotype 7 could represent old adults who exhibit extreme signs of immune remodeling and aging. On the contrary, immunotype 2, despite being the second youngest immunotype in terms of median age, showed higher HLA‐DR+ CD4+ cells than immunotypes 1, 3, 5, and 6, and lower true naive CD4+ cells than immunotypes 1, 3, and 6, which could indicate an early sign of an aging‐associated phenotype (Figure 4a,g,i).

### 2.5 Immune stability over time is a feature of immunotypes

**FIGURE 5:**
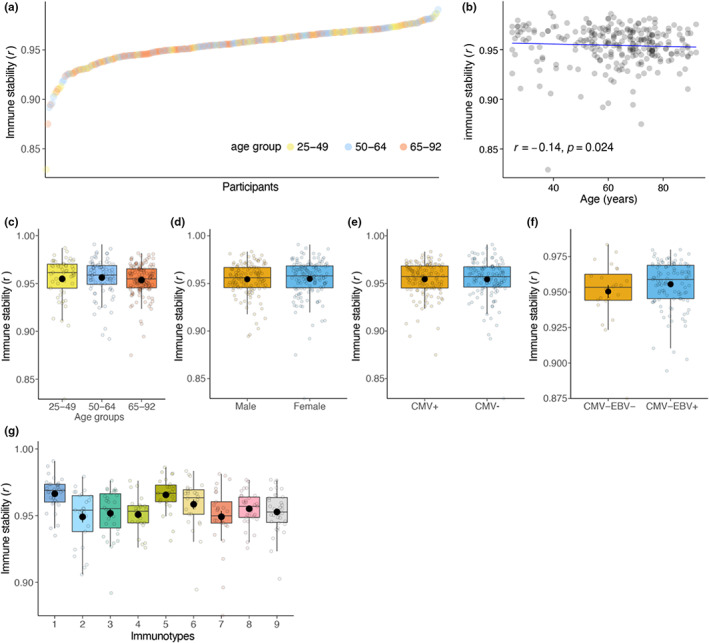
Immune stability is associated with immunotypes but not with sex, CMV‐seropositivity, and EBV‐seropositivity. (a) Intra‐individual immune subset correlation which reflects the immune stability ordered from low to high. Each point represents an individual colored by age groups they belong. (b) Spearman correlation between immune stability and age. (c–g) immune stability in age groups, sex, CMV‐seropositivity, EBV‐seropositivity, and immunotypes

As the immune system is subjected to constant remodeling and the inter‐individual variation increases with age, we hypothesized that the rate of intra‐individual variation over time could be a feature associated with the composition of the immune cell subsets. To assess this, we compared immune subset composition (percentages and absolute numbers) of each individual between Day 0 and approximately one‐year post‐vaccination to measure intra‐individual variation. Days between sampling (median = 325 days, Inter Quartile Range = 47 days) were not associated with the intra‐individual correlation (*r* = 0.005, *p* = 0.93), and previous studies showed that immune system variation is stable over the course of months to a year (Carr et al., 2016; Lakshmikanth et al., 2020; Tsang et al., 2014) (Figure S5). According to this, we observed that most individuals showed high correlation scores between Day 0 and one‐year time points; in other words, high immune stability is observed over time. However, some individuals had lower or higher stability than others (Figure 5a). We observed a weak but significant inverse correlation between age and immune stability (*r* = −0.14, *p* = 0.024, Figure 5b). To better understand this variation in immune stability, we investigated immune stability by age groups, sex, CMV‐seropositivity, and EBV‐seropositivity but did not observe any significant difference (Figure 5c–f). In contrast, immunotypes illustrated differences in immune stability. Immunotypes 1, 5, and 6, which are the least aging‐associated phenotypes, exhibited the highest immune stability scores (Figure 5g). Especially immunotypes 1 and 5 exhibited significantly high immune stability compared with immunotypes 2, 4, 7, and 9, which are the most aging‐associated phenotypes (Figure 5g, Bonferroni–Hochberg‐corrected *p*‐value<0.001). One of the most predominant features of immunotypes 1, 5, 6, which showed the highest immune stability, was a lower HLA‐DR+ CD4+ and HLA‐DR+ CD8+ T cell percentage (Figure 4a, Table S3). On the contrary, immunotypes 2, 4, 7, and 9, which showed the lowest immune stability, displayed a higher HLA‐DR+ CD4+ and HLA‐DR+ CD8+ T‐cell percentage (Figure 4a, Table S3). In summary, accelerated intra‐individual immune variation in older adults and lower immune stability in age‐associated immunotypes could provide further clues to identify at‐risk individuals in the population.

### 2.6 Immune stability correlates with circulating CD38+ CD4+ T follicular cell kinetics after influenza vaccination

**FIGURE 6:**
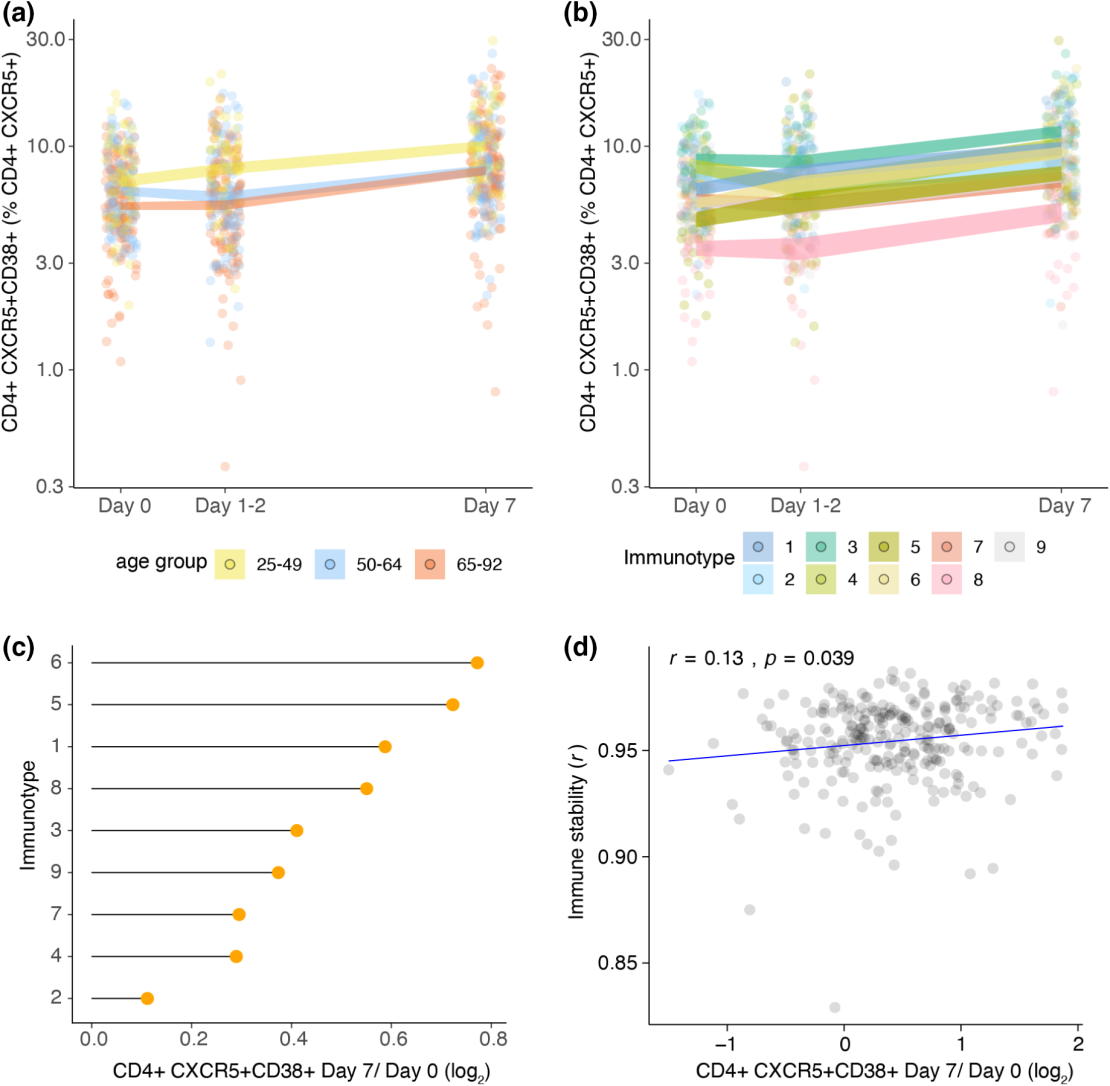
Circulating CD38+ CD4+ T follicular cell kinetics 7 days after influenza vaccination in age groups and immunotypes (a) circulating CD38+ CD4+ T follicular cells before and 7 days after influenza vaccination in age groups and (b) in immunotypes. (c) Mean CD38+ CD4+ T follicular cell log_2_ fold change between before and 7 days after influenza vaccination in immunotypes. (d) Spearman correlation between immune stability and circulating CD38+ CD4+ T follicular cell log_2_ fold change between before and 7 days after influenza vaccination

Next, we investigated whether immunotypes may associate with post‐influenza vaccination cellular kinetics. We analyzed activated (CD38+) circulating CD4+ T follicular cells pre‐ and post‐vaccination which were previously shown to be a good predictor of influenza vaccination response (Cole et al., 2019; Koutsakos et al., 2019; Pilkinton et al., 2017). In young adults, circulating CD38+ CD4+ T follicular cell percentages were higher than middle‐aged and older adults before vaccination (Figure 6a). These cells increased in percentage 7 days after vaccination in all age groups, but an early increase 1–2 days after vaccination was observed only in young adults (Figure 6a). Similar to young adults, immunotypes 1, 5, and 6, which had high immune stability scores, demonstrated an early increase 1–2 days after vaccination compared with the rest of the immunotypes (Figure 6b). The highest circulating CD38+ CD4+ T follicular cell increase between Day 0 and 7 days after vaccination was observed in immunotypes 1, 5, and 6 (Figure 6c). In contrast, immunotypes 2, 4, and 7, which had low immune stability scores, showed relatively lower circulating CD38+ CD4+ T follicular cell kinetics than immunotypes 1, 5, and 6 (Figure 6c). Circulating CD38+ and total CD4+ T follicular cell percentages and absolute numbers also displayed an increase after vaccination but these post‐vaccination kinetics were less pronounced in the total circulating CD4+ T follicular cell subset (Figure S6A–C). Moreover, immune stability correlated with circulating CD38+ CD4+ T follicular cell kinetics before and after vaccination (*r* = 0.13, *p* = 0.039, Figure 6d). These observations suggest that stability of the immune system over time could be a feature that associates with immune cell subset composition and immune activation of vaccination‐relevant cell subsets.

## 3 DISCUSSION

The human immune system is exceedingly diverse among individuals (Liston et al., 2021), and our understanding of immune variation and the association between immune variation and immune function is still limited. In this study, we were able to dissect inter‐individual immune variation into nine immunotypes that exhibited unique immune signatures and explained inter‐individual immune variation better than age alone. Moreover, we identified immunotypes of both younger and older individuals with signatures of aging‐associated immune subset compositions and CMV infection. In addition, we showed that immune stability was associated with immune immunotypes and circulating CD38+ CD4+ T follicular cell increase 7 days after influenza vaccination.

Thus far, several studies have aimed to cluster inter‐individual immune variation; however, they did not report discrete immunotypes (Kaczorowski et al., 2017; Kanodia et al., 2019; Liefferinckx et al., 2021). In these studies, characteristics of the study population, immune parameters investigated, and clustering methods could be the reasons for the lack of immunotype identification. Our study confirms previous reports that inter‐individual immune variation is associated with age and CMV‐seropositivity (Carr et al., 2016; Jergović et al., 2019; Liston et al., 2016). Therefore, age distribution and CMV‐seropositivity could determine which types of immune phenotypes are present in a study population. Next to that sample size and general health status of a population could be involved. The VITAL cohort contains individuals from a large age range with a focus on middle‐aged and older adults, which are the groups we expect to observe diverse immune phenotypes and aging‐associated remodeling of the immune system. Therefore, this cohort is especially suitable to investigate the effect of age on inter‐individual immune variation and diverse immune phenotypes. Another aspect of immune variation that we investigated extensively was the inclusion of absolute counts of immune cell subsets, which was often overlooked in other studies. Distinct immunotypes, age, and CMV‐seropositivity explained 19%, 5%, and 5% of immune variance, respectively. This means that a substantial amount of variance remained unexplained. Accordingly, despite being useful tools to describe and study immune variation, immunotypes are a representation of continuous immune variation in our cohort. A recent study investigated immune variation along one continuum using multiple omics technologies (Alpert et al., 2019). On the contrary, Alpert et al., 2019 reported additional trajectories and homeostatic states may exist, since the constructed trajectory is again an approximation of potentially multiple immune trajectories in the population (Liston et al., 2021). A previous study showed that genetic drivers could account for as much as 37% of the immune variation (Orrù et al., 2020). Another study described that non‐heritable factors explain the immune variation more than heritable factors (Brodin et al., 2015). Although we included herpes virus infection status, not all non‐heritable factors were investigated in our study.

In addition, longitudinal data collection on both percentage and absolute number of immune cell subsets in the VITAL cohort offered a unique opportunity to investigate intra‐individual immune variation. Especially in this context, we believe that it is crucial to incorporate absolute numbers of immune subsets in the analysis for two main reasons. Firstly, absolute numbers give information about the subset within the total immune cell population, whereas immune subset percentages are mostly reported as the percentage of the parent population, making it a relative factor, and therefore, it represents different characteristics of the immune subset composition. Secondly, when the absolute numbers of the subset of interest change similar to its parent population, the percentage of the immune subset may remain static. Therefore, the investigation of absolute numbers in intra‐individual variation provides additional resolution. We calculated intra‐individual correlation of immune subset composition (percentage and absolute number) over approximately a year to describe the stability of immune system. One of the most surprising results from our study was that intra‐individual immune variation, which reflects immune stability, was a feature of immunotypes. In immunotypes that exhibited signatures of aging‐associated immune subset composition, immune stability was significantly lower. A recent study measured intra‐individual immune variation in 1 year based on the immune cell frequencies and showed that a higher intra‐individual variability was associated with plasma proteins that are the markers of metabolic health (Lakshmikanth et al., 2020). Others used longitudinal data to describe immune variation in a continuum and developed a clinically meaningful metric that associates with mortality (Alpert et al., 2019). Our results showed that immune stability correlated with a circulating CD38+ CD4+ T follicular cell increase 7 days after influenza vaccination. This could indicate that immune stability is not only associated with immune phenotypes but also immune responsiveness.

We confirm previous observations by other studies that CMV‐seropositivity is associated with an increased inter‐individual immune variation (Carr et al., 2016; Jergović et al., 2019; Liston et al., 2016). We speculate that chronic CMV infection may drive this variation by forcing the immune system to remodel. Notably, CMV‐seropositivity, which has been shown to have an enormous effect on immune subsets, was not previously investigated in the context of intra‐individual immune variation. We showed that CMV‐seropositivity did not significantly influence immune stability. We hypothesize that once an individual is able to cope with the challenges CMV poses on them, the stability is unaffected. This is in line with recent studies describing that CMV‐seropositivity does not affect the immune response to influenza vaccination (van den Berg et al., 2018) or infection (van den Berg et al., 2021). These reports fit with our observation of the relationship between immune stability as a parameter of immune function and CMV‐seropositivity.

Immunotypes and immune stability could serve as novel biomarkers to help identify individuals at risk of developing low vaccination responses. This would aid in improved protection by adaptation of vaccination strategies. At the moment, our definition of immune stability requires longitudinal and complex multi‐color flow cytometry data collection with counting beads. In fact, the availability of longitudinal and immune cell subset data (percentage and absolute count) in the clinic is common, especially for individuals with chronic diseases who are amongst those with increased risk. We observed that immunotypes with significantly higher HLA‐DR+ CD4+ and lower CD95+ CD4+ T cells showed low immune stabilities. The discovery of additional markers to describe immune stability in simpler forms could potentially move this concept from bench side to bedside in the future.

More research is needed to explain the properties of immunotypes in a multi‐factorial systems approach that considers cytokine profiles, transcriptomics, microbiome, and physiological function combined with environmental exposures. In addition, the relationship between immune stability and immune function in disease and vaccination responses should be examined in more detail. We observed that age was negatively correlated with immune stability, which could mean that the immune systems of older individuals undergo more rapid modulation than younger individuals. One of the driving factors of the lower immune stability we observed in older individuals could be inflammaging, a phenomenon associated with low‐grade chronic inflammation that could accelerate immune modulation (Fülöp et al., 2019; Furman et al., 2017; Sayed et al., 2021). In immunotypes with low immune stability, we observed higher CD4+ Tregs, HLA‐DR+ CD4+ Tregs, and HLA‐DR+ CD8+ T cells, which have regulatory functions. One explanation for this observation could be the need to regulate and suppress immune activation in a chronic low grade inflammation setting. Interestingly, a recent study showed that a pro‐inflammatory cytokine environment could suppress antigen‐specific circulating T follicular cell formation after influenza vaccination (Hill et al., 2021). This is in line with our observation where immunotypes 2 and 7 with low immune stability, higher regulatory pressure and potentially inflammaging driven remodulation showed lower CD38+ CD4+ T follicular cell increase 7 days after influenza vaccination.

Overall, our study demonstrates that inter‐individual immune variation described as immunotypes could help us better understand immune variation in the aging population and identify individuals in need of improved vaccination strategies. In addition, immune stability is a feature of immunotypes that could be a potential indicator of post‐vaccination cellular kinetics and could provide opportunities to identify individuals with lower immune responsiveness.

## 4 METHODS AND MATERIALS

### 4.1 Cohort description, study subjects and sampling

The Vaccines and InfecTious diseases in the Aging popuLation (VITAL) cohort started in 2019 in the Netherlands (September 2019–October 2022) (van Baarle et al., 2020). The purpose of the study was to investigate age‐associated changes in immune functioning. To this end, individuals (*N* = 326) were recruited in the Netherlands and categorized into three age groups: young (25–49 years), middle‐aged (50–64 years), and older adults (65–92 years) (Figure S1). The young‐ and the middle‐aged individuals were employees recruited at the Dutch National Institute for Public Health and the Environment (Rijksinstituut voor Volksgezondheid en Milieu, RIVM) (Bilthoven), University Medical Center Utrecht (Utrecht), and Spaarne Hospital (Hoofddorp). The older adults were recruited from a previous study on influenza‐like‐illness in older adults (van Beek et al., 2017). Capacitated individuals of 25 years or older who have signed informed consent were included in the study. As influenza vaccination history has a considerable influence on influenza immune responses, only individuals who were vaccinated with the previous year's seasonal influenza vaccine (2018–2019) were included in the study (Tsang et al., 2014). Individuals were excluded when they use or used immune‐modulatory drugs or have a disease that make them immunocompromised, including recipient of an organ‐ or bone marrow transplant, used high‐dose of daily corticosteroids or received chemotherapy in the last 3 years (Figure S1). In addition, individuals were excluded when they had a history of allergic reaction to vaccine components and factors that may interfere with blood collection, including known anemia (hemoglobin 8.5 mmol/L for men, 7.5 mmol/L for women) and known or suspected coagulation disorder, or immunological analyses. Whole blood samples were collected from these individuals for immune phenotyping before (Day 0), 1–2 days, 7 days, and approximately 1 year after vaccination with the 2019–2020 seasonal quadrivalent inactivated influenza vaccine (Figure 1). Whole blood samples were collected in EDTA pre‐coated tubes. The study was approved by Medical Research Ethics Committee Utrecht (EudraCT Number: 2019–000836–24) (Anon Clinical Trials register n.d.). This study was performed according to Good Clinical Practice, the Declaration of Helsinki, and written informed consent was obtained from all participants.

### 4.2 Hematology analysis and flow cytometry immunotyping

Whole blood samples were analyzed the same day of blood withdrawal on a DxH 500 Hematology Analyzer (Beckman Coulter) to assess the absolute number and percentages of leucocytes (*N* = 301). Next, whole blood samples were analyzed for expression of specific immune markers to further characterize immune cell subsets. To this end, whole blood samples (*N* = 318) were stained using the following anti‐human fluorochrome‐conjugated antibodies: CD45 (HI30, Biolegend), CD3 (UCHT1, Biolegend), CD4 (SK3, BD Biosciences), CD8 (RPA‐T8, Biolegend), CD19 (SJ25C1, BD Biosciences), CD14 (HCD14), CD56 (NCAM16.2), CD16 (3G8), CD27 (O323), CCR7 (G043H7), CD45RO (UCHL1, Biolegend), CD95 (DX2, Biolegend), CD38 (HIT2, BD Biosciences), CXCR5 (RF8B2, BD Biosciences), CD25 (2A3, BD Biosciences), CD127 (A019D5, Biolegend), IgD (IA6‐2, Biolegend), HLA‐DR (G46‐6, BD Biosciences). Samples were stained in TruCOUNT tubes (BD Biosciences) at room temperature for 15 min in the dark. According to the manufacturer's lyse‐no‐wash protocol, red blood cells were lysed and samples were acquired on a 4‐laser LSRII Fortessa X20 flow cytometer (BD Biosciences). Cell subset gating was performed in FlowJo (V10.7.1, Tree Star) (Figure S7). Cell subsets with less than 20 events count on average were removed from the analysis. According to the manufacturer's protocol, absolute cell counts were calculated from event counts of TruCOUNT beads. Downstream analyses were performed in R studio (v1.3.1073). Subsets that were measured by both hematology and flow cytometry immunotyping assays showed high correlations (Spearman's rho>0.8, *p* < 2.2e‐16 for monocyte and lymphocyte absolute number and percentages).

### 4.3 Inter‐individual and intra‐individual immune variation

To study the inter‐individual immune variation between the groups (age group, sex, CMV‐seropositivity, and EBV‐seropositivity), we calculated the intra‐group Spearman correlation between individuals based on the percentages and absolute numbers of their immune cell subsets. To study the intra‐individual variation, we calculated Spearman correlation per individual between pre‐vaccination (Day 0) and approximately one‐year timepoint (median = 325 days, Inter Quartile Range = 47 days), based on percentages and absolute numbers of immune cell subsets.

### 4.4 Principal component and clustering analyses

Immune cell subsets are hierarchal in their nature and since immune cell subsets are studied as subsets of parent populations, these parameters are often overlap with each other, that is, represent similar information. Therefore, we rationally selected 59 immune cell subsets with minimal overlap to represent the diverse characteristics (percentages of innate immune subsets, adaptive immune subsets, naive, memory, activated, regulatory) of the immune system subsets (Table S1). We calculated Cook's distance (Cook, 1977) based on main immune cell subset percentages (CD19+, CD3+, CD3‐, CD4+, CD8+, granulocytes, lymphocytes, and monocytes). Extreme outliers (individuals with Cook's distance cut off>15) were removed from the principal component and clustering analyses based on these parameters. Next, we generated a correlation matrix of individuals by calculating pairwise Spearman's correlation coefficients between individuals based on the 59 immune cell subsets. Using Gap statistics (Tibshirani et al., 2001), we calculated the optimal number of clusters in the Spearman matrix of individuals and clustered the data using k‐means (Hartigan & Wong, 1979) clustering.

### 4.5 Cytomegalovirus and Epstein–Barr virus seropositivity

Immunoglobulin G antibodies against CMV and EBV were quantified in serum collected before vaccination by a multiplex immunoassay developed in‐house (Korndewal et al., 2015). Seropositivity thresholds were adapted from a previous study (Samson et al., 2020). For CMV, a concentration of <4 relative units (RU) ml^−1^ was categorized as seronegative, ≥4 and <7.5 RU ml^−1^ as borderline, and ≥7.5 RU ml^−1^ as seropositive. For EBV, a concentration of <16 RU ml^−1^ was categorized as seronegative, ≥16 AU ml^−1^, and <22 RU ml^−1^ as borderline and ≥ 22 RU ml^−1^ as seropositive.

### 4.6 Statistical analysis

R (v4.0.2) and Rstudio (v1.3.1073) were used for the analyses. For Cook's distance calculation car (v3.0–11), for principal component analysis (data were scaled) FactoMineR (v2.4), for Gap statistics (k.max = 20, nboot = 50, nstart = 25) factoextra (v1.0.7), for heatmaps ComplexHeatmaps (v2.6.2), for Permutational analysis of variance analysis (method = “canberra,” permutations = 1000) vegan (v2.5–7), for statistical analyses rstatix (v0.7), for k‐means clustering (nstart = 100) stat (v4.0.2) packages were used. Statistical significance is indicated in figures as follows: *p < 0.05, **p < 0.01, ***p < 0.001, ****p < 0.0001.

## AUTHOR CONTRIBUTIONS

AC, SAS, NR, AMB, and DvB conceptualized the study. AC, SAS, and NMN performed methodology. AC, SAS, MV, and NMN performed the study. AC and SAS visualized the data. NR, AMB, and DvB were involved in funding acquisition. LB, EB, JvB, and DvB were involved in project administration. SAS, AMB, and DvB supervised the study.

AC wrote the original draft. AC, SAS, AMB, DvB involved in writing—review and editing.

## FUNDING INFORMATION

The VITAL project has received funding from the Innovative Medicines Initiative 2 Joint Undertaking (JU) under grant agreement No. 806776 and the Dutch Ministry of Health, Welfare and Sport. The JU receives support from the European Union's Horizon 2020 research and innovation programme and EFPIA‐members.

## CONFLICT OF INTEREST

None declared

## Supporting information


Table S1

Table S2

Table S3

Figures S1–S7

## Data Availability

Research data are not shared since the pre‐clinical trial is ongoing and the primary endpoints are not yet published
