# Supplementary material for: Identification of aging‐associated immunotypes and immune stability as indicators of post‐vaccination immune activation
Source: Aging Cell. 2022 Sep 8;21(10):e13703. doi: 10.1111/acel.13703 (PMC9577949; doi:10.1111/acel.13703)
Supplement: Supplementary file 4 — Figures S1–S7 [file ACEL-21-e13703-s002.docx]

**
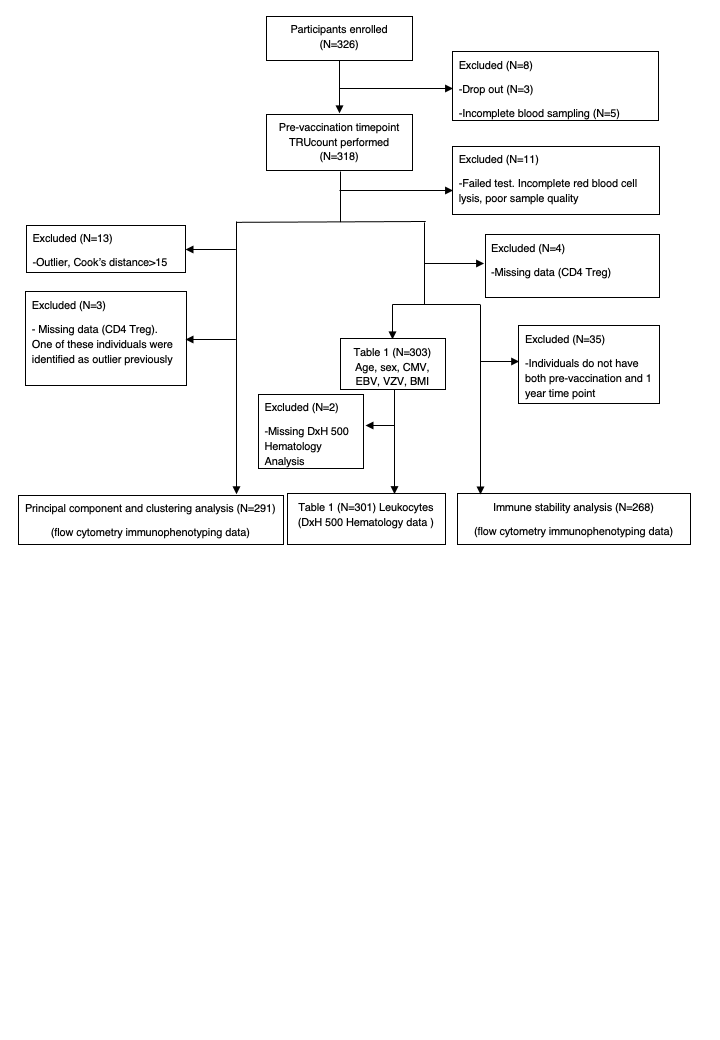
**

**Supplementary Figure 1:** Flow chart of data analyses.

**
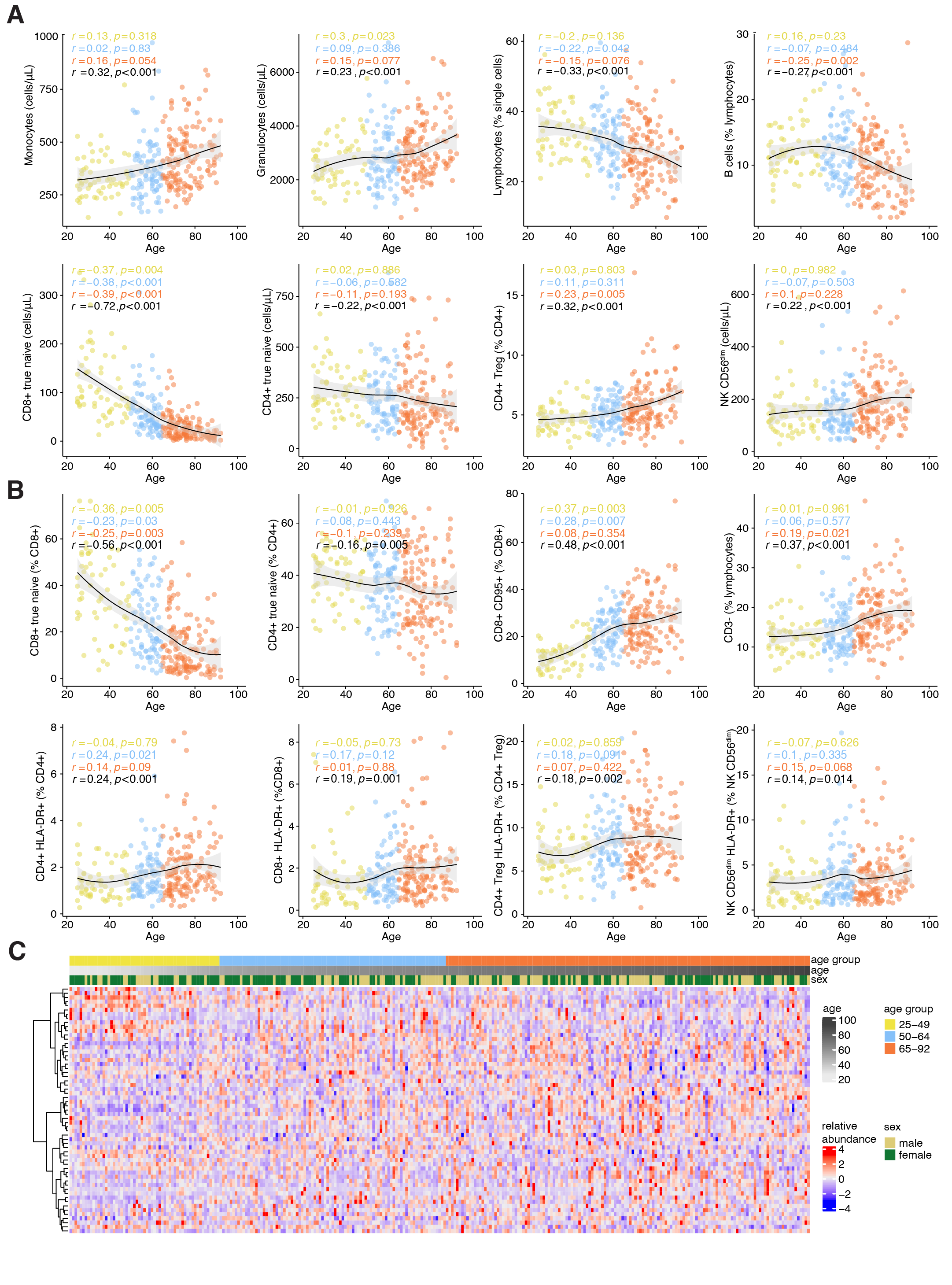
Supplementary Figure 2:** **(A,B)** Spearman correlation between selected immune cell subsets and age in young adults, middle-aged adults and older adults. Coloured *r* shows the correlations for the corresponding age group, r values in black show the correlations in all individuals The area around the loess fitted regression line describes 95% confidence interval the curve described 95% confidence interval. **(C)** Heatmap of 59 immune cell subset percentages (rows) for each individual (columns). Individuals are ordered by increasing age from left to right.


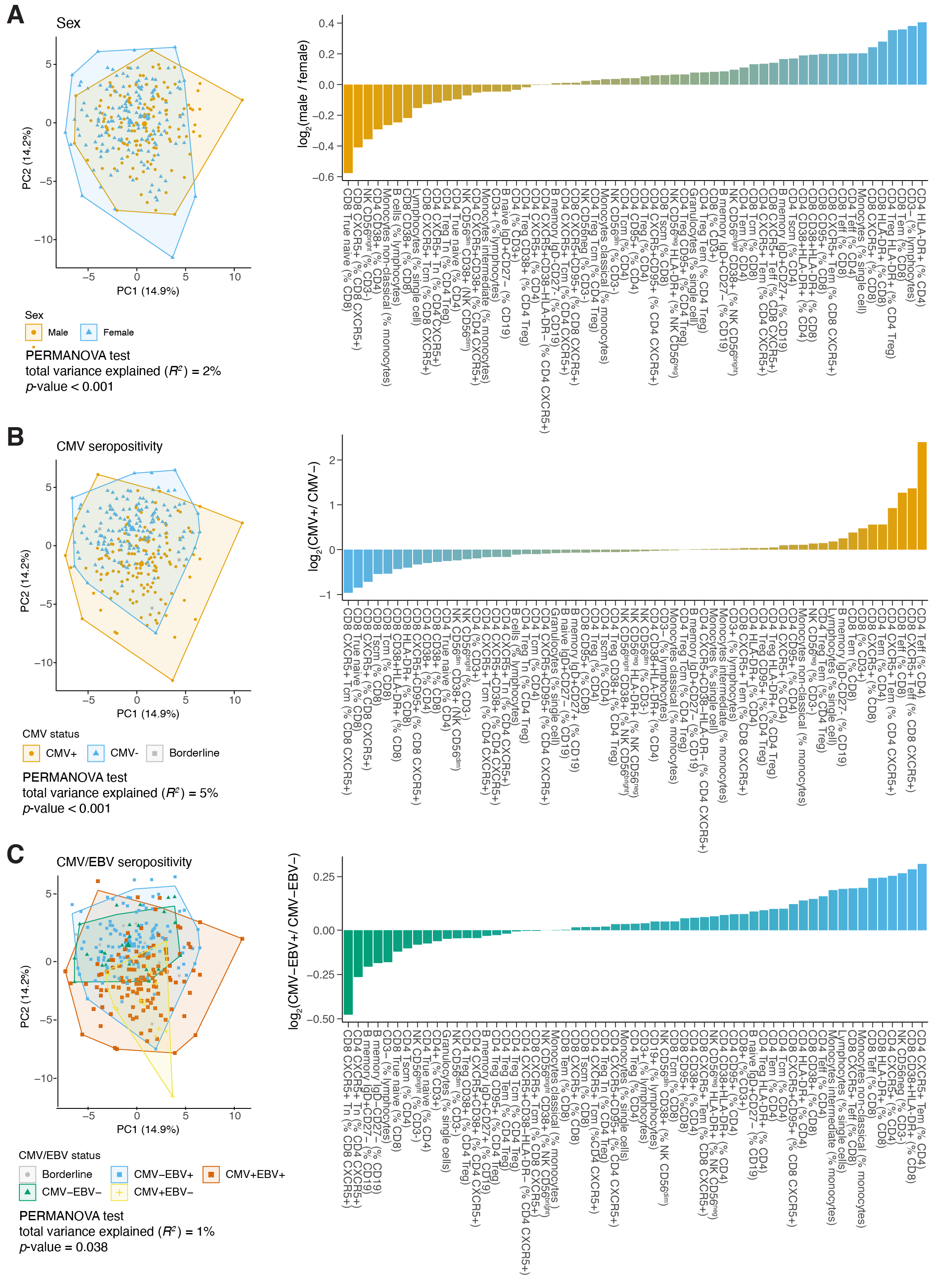


**Supplementary Figure 3:** Annotation of individuals on PC analysis for **(A)** sex, **(B)** CMV-seropositivity and **(C)** EBV-seropositivity. Bar plots represent percentages of 59 immune cell subset used in PC analysis as log_2_ ratios between each group investigated. Borderline cases for CMV and EBV were removed from PERMAVONA analyses.


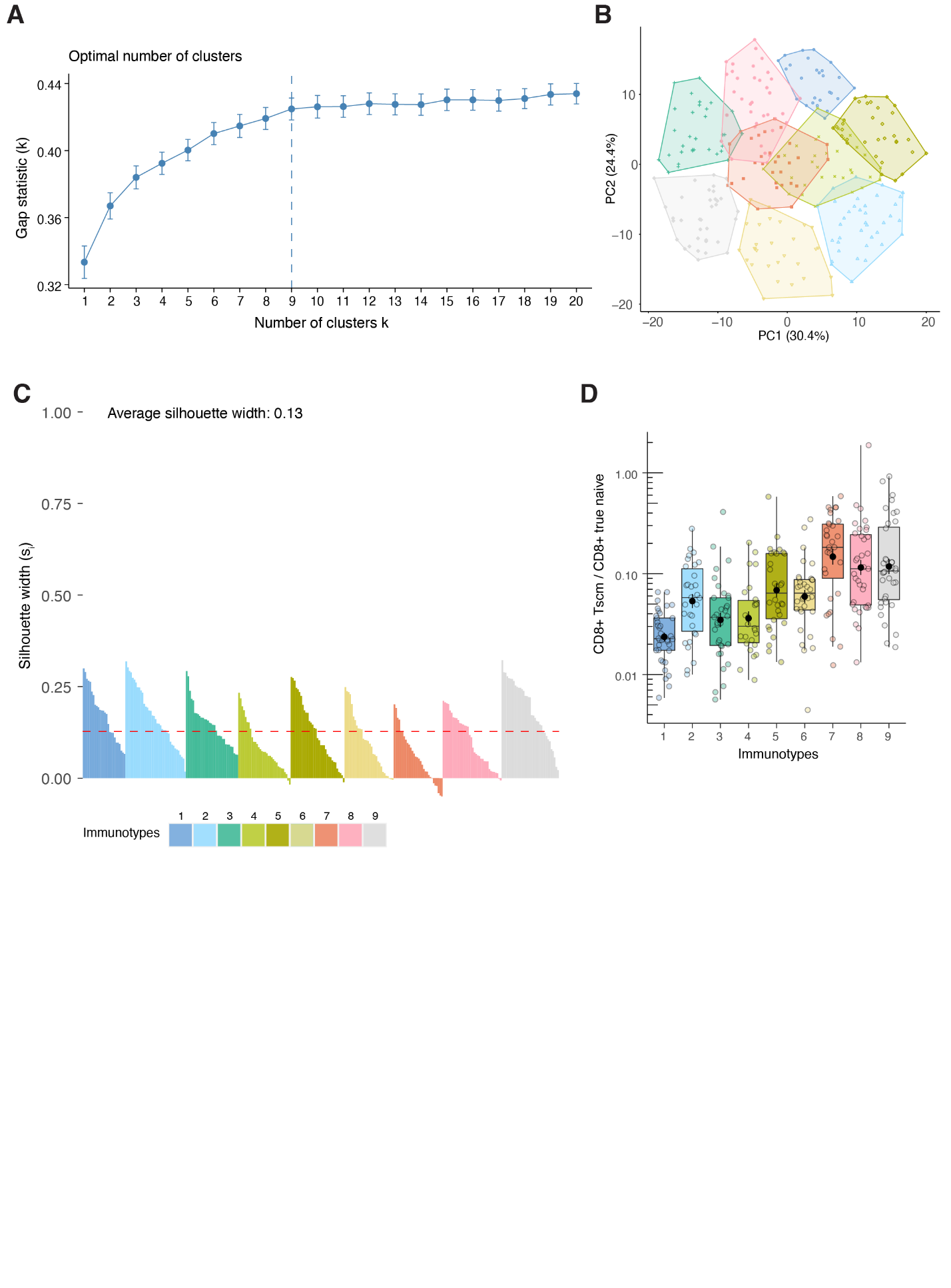


**Supplementary Figure 4:** **(A)** Gap statistics showing the optimal number of clusters/immunotypes. Gap statistics applied on the Spearman correlation matrix of individuals that was calculated based on percentages of 59 immune cell subsets. **(B)** Representation of immunotypes on PCs based on Spearman correlation matrix of individuals. **(C)** Silhouette plot illustrating Silhouette value (s_i_) for each cluster/immunotype. The average silhouette width is marked with the dotted red line. **(D)** CD8+ T stem cell like memory (Tscm) /CD8+ true naïve ratio in immunotypes (Dunn’s test, Benjamini-Hochberg corrected *p*-values).


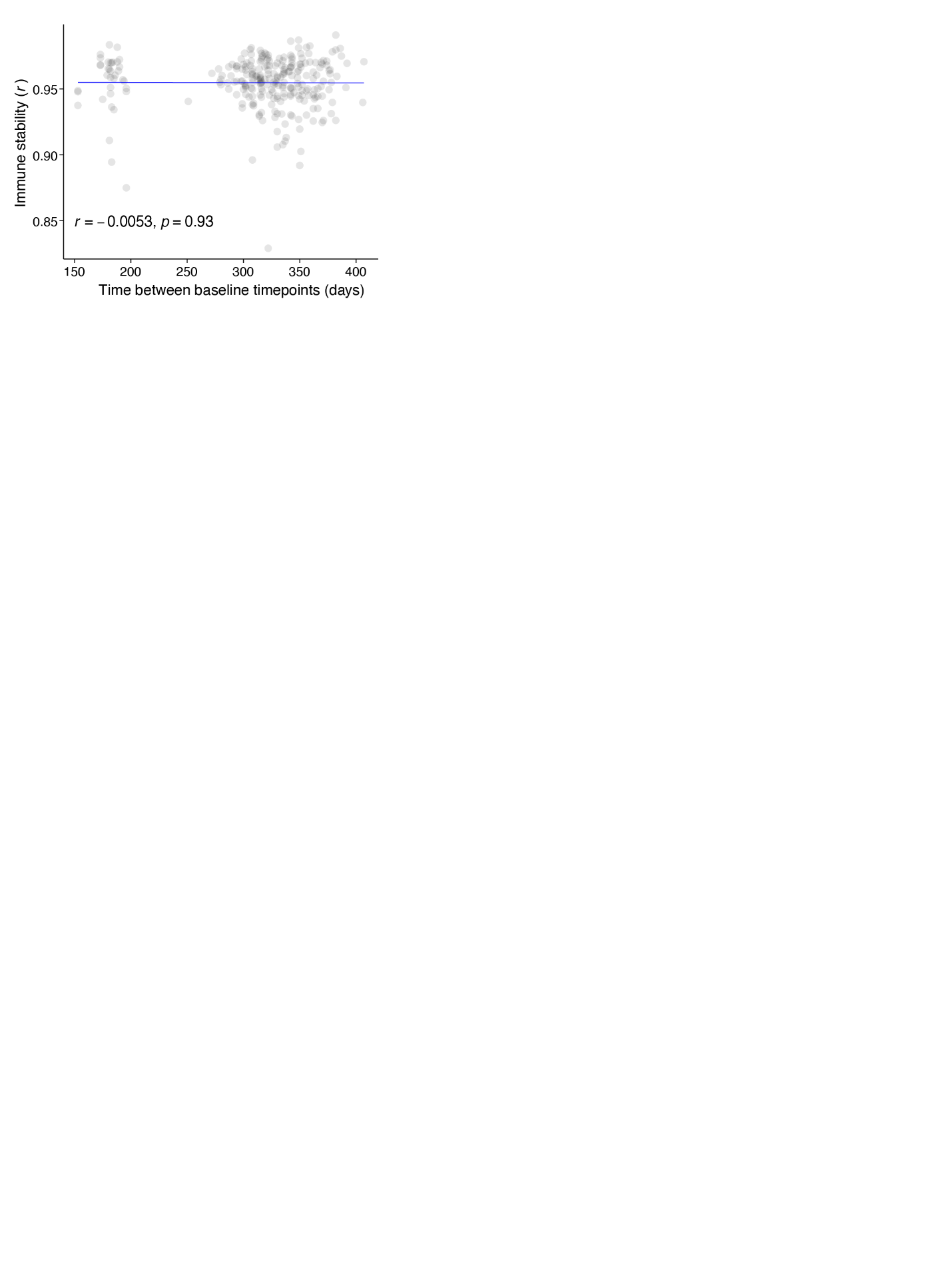


**Supplementary Figure 5:** Spearman correlation between immune stability and time between day 0 and approximately one year time point.


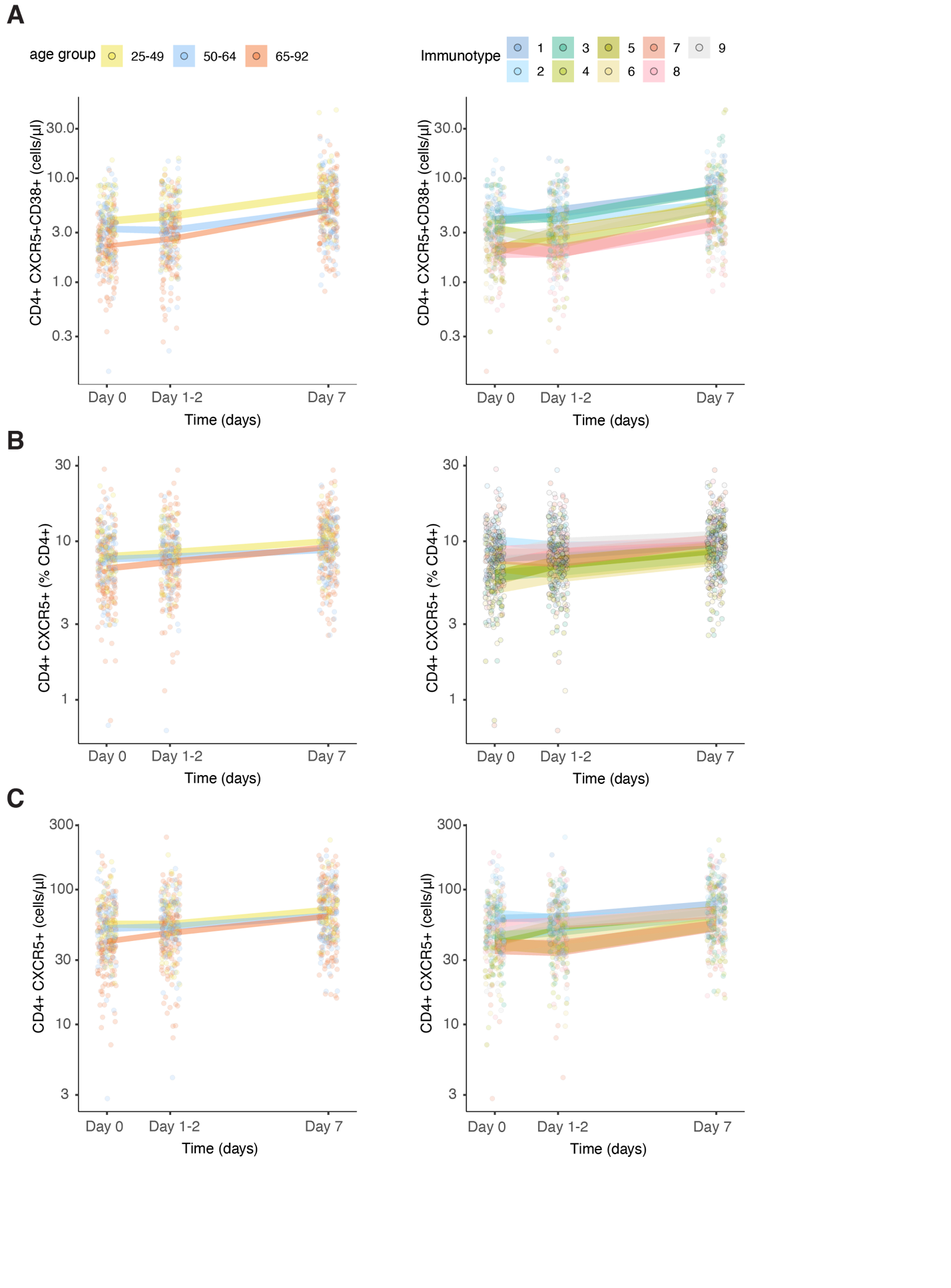
**Supplementary Figure 6**: Circulating (CD38+) CD4+ T follicular cell kinetics 7 days after influenza vaccination in age groups and immunotypes. **A)** CD4+ CXCR5+ CD38+ (cells/ul) **B)** CD4+ CXCR5+ (% CD4+) **C)** CD4+ CXCR5+ (cells/ul)


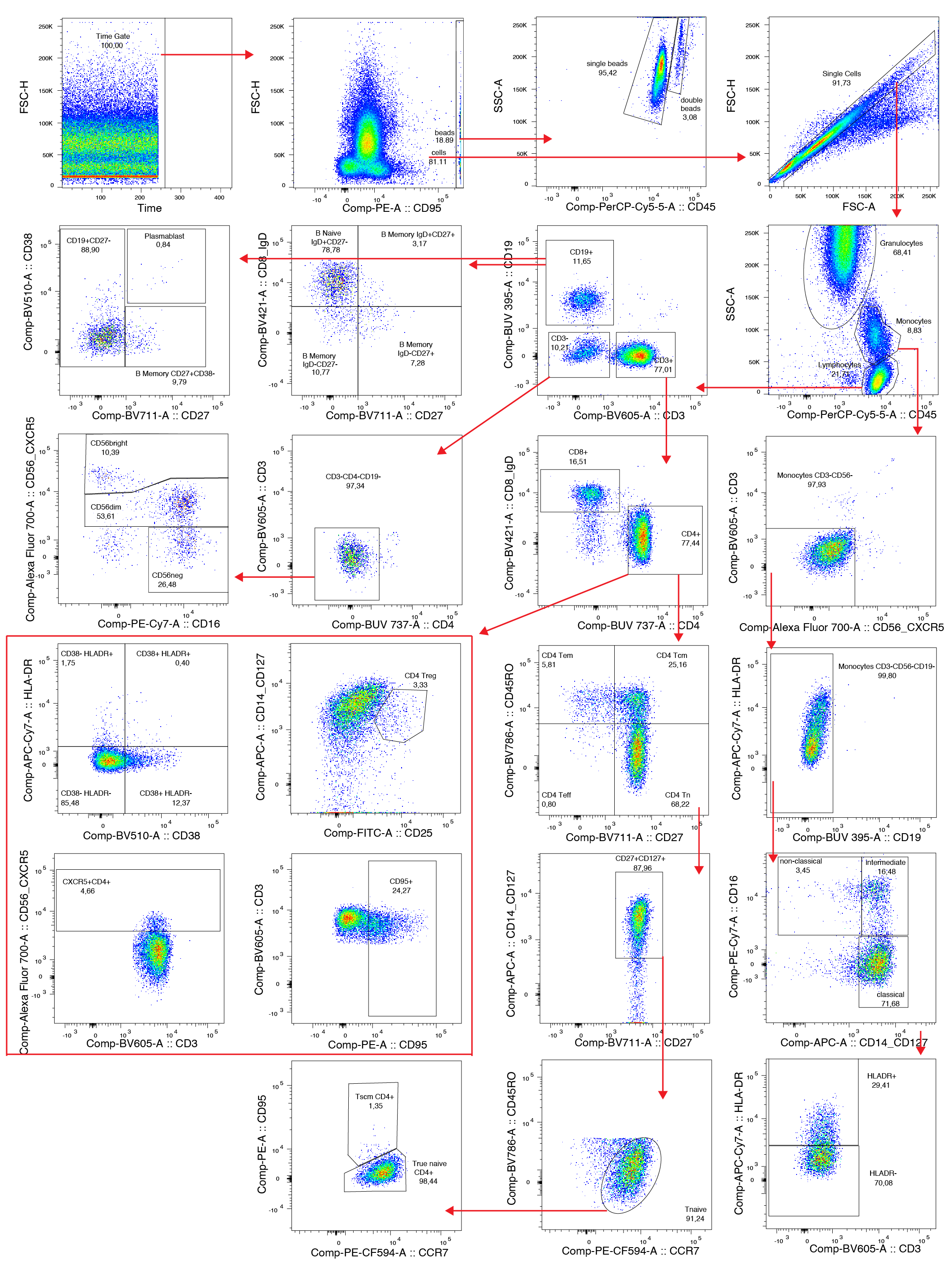


**Supplementary Figure 7:** Gating strategy of immune cell subsets. For T cells only CD4+ gates are depicted. Same gates apply to CD8+ T cells.

**Table S1, Spearman correlation analysis between age and residuals of linear regression for immune cell subsets (immune subsets ~ age, sex, CMV-seropositivity)** (in excel format)

**Table S2, Immune subset correlations with age & immune subsets used in PCA & Clustering** (in excel format)

**Table S3, significant immune subset differences between immunotypes** (in excel format)
